# Cross‐cultural differences in depression between White British and South Asians: Causal attributions, stigma by association, discriminatory potential

**DOI:** 10.1111/papt.12428

**Published:** 2022-10-27

**Authors:** Michèle D. Birtel, Briana L. Mitchell

**Affiliations:** ^1^ School of Human Sciences, Institute for Lifecourse Development University of Greenwich London UK

**Keywords:** causal attributions, cultural differences, depression, mental health, stigma

## Abstract

**Objectives:**

Numerous facets of public and internalized mental illness stigma have been established. This study focuses on the stigma of being associated with someone with depression and cultural differences between a Western and an Eastern culture. The aim was to compare White British and South Asians living in the United Kingdom regarding their causal explanations for depression, stigma towards people with depression and stigma by association.

**Design:**

A cross‐sectional design.

**Methods:**

White British and South Asians (*N* = 137) in the United Kingdom completed a survey measuring attributions about the aetiology of depression, discriminatory potential towards people with depression and stigma by association.

**Results:**

Results revealed that South Asians attributed greater supernatural, moral and psychosocial causes to depression, while White British endorsed greater biological beliefs. South Asians reported a greater discriminatory potential towards people with depression (lower willingness for closeness, greater desire for social distance) than White British. They also indicated greater affective, cognitive and behavioural stigma by association. Stigma by association mediated the relationship between cultural group and willingness for closeness as well as desire for social distance. Perceived dangerousness was a mediator for willingness for closeness.

**Conclusions:**

These findings suggest that a greater consideration of the role of culture in the understanding of mental health is important to combat stigma towards individuals with depression and those close to them across Western and Eastern cultures.

**Practitioner Points**

- Social consequences of stigma for the individual and individuals associated with them (negative feelings and beliefs, avoidance) are greater for South Asians than for White British.
- Stigma by association and perceived dangerousness mediate the link between cultural group and discriminatory potential.
- To maximize help‐seeking in people with depression and support their family members, interventions need to address stigma and stigma by association.
- Clinical approaches need to consider the role of culture in depression and stigma.

## BACKGROUND

Depression is a predominant mental illness in the world (Vos et al., 2015) and the leading cause of disability and suicide (World Health Organization, 2017). It is common in individuals who have experienced physical illnesses such as cancer, stroke and heart attack (National Institute of Mental Health, 2021). Individuals with mental illness (such as depression and schizophrenia) experience severe stigma associated with their diagnosis from the public, especially as compared to physical illness (such as cancer and heart disease; Barry et al., 2014; Corrigan et al., 2003; Crisp et al., 2000; Huggett et al., 2018; Wood et al., 2014). Cultures differ in their expression of symptoms, in Eastern cultures there is a greater emphasis on reporting physical rather than psychological symptoms (such as pain, fatigue), and stigma appears to be higher than in Western cultures (Ahmed et al., 2020; Krendl & Pescosolido, 2020; Lauber & Rössler, 2007; Mirza et al., 2019).

While previous research has established various facets of public and internalized mental illness stigma in the Western world (Rüsch et al., 2005; Thornicroft et al., 2007), cultural differences in perceptions of mental illness between Western and Eastern cultures are less well understood, particularly for stigma by association. Mental illness and stigma should be studied in its socio‐cultural context, because findings from Western cultures cannot be generalized to Eastern cultures or minority groups from Eastern cultures living in a Western dominant host culture such as South Asians in the UK. Furthermore, research that considers the role of culture has focused on public stigma rather than stigma by association. To fill the empirical gaps and contribute to a culture‐sensitive understanding of mental illness and its associated stigma, the aim of the present study was to (1) compare White British with South Asians living in the UK as a minority group in a dominant Western society, (2) examine cultural differences in regard to the attribution of the aetiology of depression as well as in public stigma towards people with depression and (3) shed light on the stigmatization process by including the stigma of being associated with a person with depression.

### Stigma towards depression

Previous literature has identified that depression is associated with more negative stereotypes than anxiety, and individuals with depression are perceived as lazy and difficult to communicate with (Thornicroft et al., 2007; Wood et al., 2014). Furthermore, greater blame is attributed to individuals with depression than schizophrenia. Those with depression are perceived as having themselves to blame, as being different, and as being able to pull themselves together (Wood et al., 2014). Stigma has been described a discrediting attribute which devalues a person's social identity (Crocker et al., 1998; Goffman, 1963). It has devastating consequences for their quality of life (Corrigan & Watson, 2002; Link et al., 1997, 2001; Perlick et al., 2001), and impacts negatively on people associated with them such as family, friends and caregivers (Corrigan & Miller, 2004; Onwumere et al., 2016; Phelan et al., 1998). Stigma theories such as Link's modified labelling theory (Link et al., 1989; Link & Phelan, 2001) suggest that the process of receiving a diagnosis and entering treatment attaches a label onto the individual which then evokes stigmatizing reactions from the public and health professionals. Furthermore, having a mental illness, or being associated with someone with a mental illness, could be considered as possessing a social identity that is devalued by others (Crocker et al., 1998). A devalued social identity can feel threatening, according to Major and O'Brien's (2005) stigma‐induced identity threat model, and experiencing identity threat can negatively affect people's mental and physical health.

### Cross‐cultural differences in depression

Stigma towards mental illness appears to be higher in Eastern than in Western cultures (Krendl & Pescosolido, 2020; Lauber & Rössler, 2007), for example towards individuals with psychosis (Ahmed et al., 2020; Mirza et al., 2019). Different factors may contribute to higher stigma and less help‐seeking in Eastern cultures, such as cultural values emphasizing relationships, attributing mental illness to different causes and the reduced distinction between mind and body.

#### Causal attributions

In Eastern cultures, beliefs about the aetiology of mental illness refer to supernatural causes (Lauber & Rössler, 2007; Mirza et al., 2019), and moral reasons such as bad character (Krendl & Pescosolido, 2020) or bad behaviour from a previous life (Raguram et al., 2004). For example, qualitative studies on psychosis indicated that South Asians in India (Saravanan et al., 2007), South Asians in the UK (Cinnirella & Loewenthal, 1999), and Indian immigrants in the UK (Jobanputra & Furnham, 2005) reported greater beliefs in supernatural causes of psychosis and religious strategies for treatment. In comparison, White British are more likely to endorse biological causes (Dein & Bhui, 2013). Culture‐specific understandings of mental illness, for example causal attributions in form of supernatural beliefs, might delay treatment, alter the type of help (medical, spiritual) people seek as well as treatment outcomes (such as attitudes towards treatment, satisfaction with the relationship with a therapist) (Burns et al., 2011; Carter et al., 2018; Islam et al., 2015).

#### Physical and mental health

In Western cultures, there is a greater distinction between mind and body, whereas such differentiation between physical and psychological symptoms is less observed in Eastern cultures (Wheeler, 1998). Psychological symptoms are expressed through physical symptoms, in other words, mental health is somatised and medicalized (Lauber & Rössler, 2007; Ng, 1997). Therefore, help‐seeking focuses on seeking help for physical distress or from a spiritual healer rather than a medical doctor or psychologist (Sanchez & Gaw, 2007).

#### Stigma by association

Stigma by association (Mehta & Farina, 1988), also termed courtesy stigma (Goffman, 1963), is the prejudice, stereotypes and discrimination experienced by stigmatized individuals that extends to those who are associated with an individual who is target of public stigma (Angermeyer et al., 2003; Corrigan & Miller, 2004; Phelan et al., 1998) and can have a similar effect to primary stigma, and even more so in Eastern cultures. Additionally, parents experience self‐stigma in regard to their perceived failure to be a good parent (Eaton et al., 2016), and family members can experience social exclusion and negative treatment. Thus, they may conceal their association and tend to socially avoid individuals with mental illness (Larson & Corrigan, 2008; Phelan et al., 1998; Shibre et al., 2001; Struening et al., 2001). Stigma by association can also reduce family functioning and closeness (Crowe & Lyness, 2014; Kreisman & Joy, 1974; Östman & Kjellin, 2002; Wahl & Harman, 1989). Similar to primary public stigma, it can have a serious impact on the quality of life of relatives, leading to poorer mental health (such as psychological distress, lower self‐esteem, anger, anxiety) and physical health (such as insomnia, fatigue and pains; Angermeyer et al., 2001, 2003; Byrne, 2000; Corrigan & Miller, 2004; Phelan et al., 1998; Wahl & Harman, 1989). To avoid the process of stigmatization, those related to people with mental illness prefer less closeness and greater social distance (Pryor et al., 2012). A cross‐sectional survey with 527 family members in the Netherlands revealed that greater perceived public stigma was associated with greater stigma by association, and in return with lower perceived closeness with a family member with a mental illness and greater psychological distress (van der Sanden et al., 2013).

In Eastern cultures stigma can have a greater impact on the individual as well as their friends and family due to the cultural emphasis on harmonious, close relationships contrary to Western cultures (Lauber & Rössler, 2007; Ng, 1997; Phillips et al., 2002; Yang et al., 2007). Previous research suggests that stigma from close others is greater in Eastern cultures and entails more consequences regarding social distance and avoidance of close relationships (Lauber & Rössler, 2007; Tabassum et al., 2000). Stigma correlates with Asian cultural values (Miville & Constantine, 2007; Shea & Yeh, 2008). For example, He and Williams (2021) demonstrated that stigma by association (in a non‐health context) can be greater in a cultural context characterized by interdependent self‐construals such as Asian and Chinese.

### Mental illness stigma in immigrants

There is a need to include the role of culture in mental health to increase the cultural sensitivity of mental health services, as clinicians report little experience and training in treating different cultures (Islam et al., 2015). Findings from other countries cannot be generalized to all Eastern countries, and findings from South Asians in their home country cannot be generalized to South Asians living in a White dominant culture such as the UK. In such a context, South Asians not only are a minority group, but they also live in a different normative environment. They may not only experience stigma due to mental illness but also due to their ethnic minority status (Major & O'Brien, 2005). They also face the challenge of acculturation processes, negotiating two cultures and adapting to new social norms (Berry, 1997, 2003; Sam & Berry, 2010). This process can induce acculturative stress (such as anxiety and depression), particularly in immigrants who are older or who have little social support. The strategies used and the outcome of the acculturation process has implications for psychological well‐being and mental health (Berry, 1997), integration and maintenance of original and host culture is the most positive outcome (Sam & Berry, 2010).

There is evidence of cultural differences between Western and Eastern cultures in causal explanations and stigma for minority groups living in a dominant society. However, most of the studies are qualitative or carried out outside the UK. For example, for depression, studies in Canada (Shamblaw et al., 2015) and the USA (Cheng, 2015; Fogel & Ford, 2005) suggested that stigma towards depression is greater for Asian Canadians and Americans than for European Canadians and Americans. Therefore, we examined South Asians as the UK's largest ethnic minority group (Office for National Statistics, 2011), and who are less likely to access mental health services than the general population in the UK (McManus et al., 2016). There is evidence that South Asians living in the UK endorse greater supernatural causes towards psychosis and greater stigma towards psychosis than White British (Ahmed et al., 2020; Mirza et al., 2019). Therefore, we predicted that this is also the case for depression. We also know that Eastern cultures such as Bangladeshi endorse greater moral causes of mental illness than Western culture such as Great Britain (Krendl & Pescosolido, 2020). Therefore, we predicted that South Asians living in the UK would endorse greater moral causes of depression than White British. Previous research has focused on public stigma when examining cultural differences, we included the stigma of being associated with a person with depression. Knowing that stigma by association mediated the relationship between public stigma and social distance in a Dutch sample (van der Sanden et al., 2013) and that perceptions of dangerousness and stigma from relatives has larger implications for social distance in Eastern cultures (He & Williams, 2021; Lauber & Rössler, 2007), we predicted perceptions of dangerousness and stigma by association to be mediators between cultural group and public stigma to shed light on the stigmatization process.

### The present research

The aim of the present research was to compare White British with South Asians living in the UK as a minority group in a dominant Western society and examine causal attributions of depression, public stigma towards people with depression and stigma of being associated with a person with depression. Beliefs about the aetiology of depression were measured using biological, psychosocial, moral and supernatural causal attributions (Krendl & Pescosolido, 2020). Stigma has been conceptualized as having three dimensions, in both Western and Eastern cultures: affective, cognitive and behavioural (Ahmed et al., 2020; Corrigan & Watson, 2002; Pescosolido & Martin, 2015; Thornicroft et al., 2007). Affective stigma refers to negative emotional responses, cognitive stigma refers to false beliefs such as perceptions of dangerousness, and behavioural stigma refers to discrimination such as exclusion and social distance. Social distance is a commonly used measure to indicate the potential to discriminate (Krendl & Pescosolido, 2020; Link et al., 1999). Therefore, as public stigma measures, we used perception of dangerousness as well as willingness for closeness and desire for social distance to measure discriminatory potential. Stigma by association was measured on all three (affective, cognitive, behavioural) dimensions. We tested the following hypotheses:

*H1*: South Asians endorse greater beliefs in spiritual (God's will) as well as moral (bad character) causes of depression, while White British endorse greater beliefs in biological (chemical imbalance in the brain, genetics) and psychosocial (upbringing, stress) causes of depression.

*H2a*: South Asians report greater perceptions of dangerousness of people with depression than White British.

*H2b*: South Asians report a greater discriminatory potential of people with depression (lower willingness for closeness and greater desire for social distance) than White British.

*H2c*: South Asians report greater affective, cognitive and behavioural stigma by association than White British.

*H3*: Perceptions of dangerousness and stigma by association mediate the association between cultural group and discriminatory potential.

## METHOD

### Participants

Participants were 91 White British (56 female and 35 male) and 46 South Asians (22 female, 23 male and one preferred not to say) in the UK (*N*_total_ = 137). White British participants had a mean age of 31.69 years (*SD* = 11.13, range 18–60), South Asian participants had a mean age of 28.96 years (*SD* = 7.62, range 18–49). An online survey on Qualtrics was distributed on social media as well as on Prolific. The inclusion criteria were being at least 18 years old and to self‐identify as either White British or South Asian. The local institutional ethics committee approved the study.

### Procedure

A vignette^1^ was presented to participants which provided them with a description of a person who meets the Diagnostic and Statistical Manual of Mental Disorders (DSM) criteria for major depression (adapted from Link et al. (1999) using the DSM‐IV). This vignette described a person named ‘Dev’ as experiencing more than five symptoms of major depressive disorder for about a month. Following this vignette, participants received scales relating to the person with depression in the vignette (identification of mental illness, causal attributions, perception of dangerousness) and scales relating to people with depression in general (social distance, stigma by association).^2^

### Measures

The scales relating to the vignette were adapted from Link et al. (1999) and assessed on a 5‐point Likert scale ranging from 1 = *very unlikely* to 5 = *very likely*. The scales were computed by the mean of the items and were reliable as indicated by Cronbach's *α*. Higher scores represented higher levels of the concepts measured.

#### Identification of mental illness

Participants were asked to identify whether the person in the vignette is experiencing a mental health problem on two items. They rated how likely it is that the person in the vignette is experiencing a mental illness, and how likely it is that the person is experiencing major depression.

#### Causal attributions

Participants were then presented with six items describing possible causes of mental illness and rated how likely each cause is for the depression of the person in the vignette. The causes related to biological causes (‘A chemical imbalance in the brain’, ‘A genetic or inherited problem’), moral causes (‘The person's own bad character’), psychosocial causes (‘Stressful circumstances in the person's life’, ‘The way the person was raised’) and spiritual causes (‘God's will’).

#### Perception of dangerousness

Participants rated how likely it is that the person with depression in the vignette would do something violent towards other people.

#### Willingness for closeness

The willingness for closeness to the person with depression in the vignette was measured by five items, for example ‘Move next door to Dev’, ‘Spend an evening hanging out or socializing with Dev’ (*α*_White_ = .92, *α*_Asian_ = .81).

#### Desire for social distance

Participants indicated how much they desire social distance from people with depression on five items (adapted from Stuart & Arboleda‐Florez, 2001), for example ‘I would be unable to maintain a friendship with someone who has depression’, ‘I would feel upset or disturbed about rooming with someone who has depression’, ranging from 1 = *disagree* to 7 = *agree* (*α*_White_ = .86, *α*_Asian_ = .85).

#### Stigma by association

Stigma by association was measured by 27 items (van der Sanden et al., 2013), ranging from 1 = *strongly disagree* to 9 = *strongly agree*. The scale captures people's affective, cognitive and behavioural reactions to being related to someone with depression.

For *affective* stigma by association (common question stem ‘How do/would you feel about having a family member who has depression?’), participants reported their feelings on 11 items (common answer stem ‘I would feel’), for example 'ashamed', 'embarrassed', 'humiliated' (*α*_White_ = .79, *α*_Asian_ = .90). For *cognitive* stigma by association (common question stem ‘What are your beliefs about having a family member with depression?’), participants reported their beliefs on seven items, for example ‘People might look down on me if they discover that I am related to this person’, ‘People would probably think that I am strange if they discover I am related to this person’ (*α*_White_ = .95, *α*_Asian_ = .97). For *behavioural* stigma by association (common stem ‘What would be your behavioural reactions about having a family member with depression?’), participants reported their behaviours on nine items, for example ‘I would not introduce my friends to this person’, ‘When the person with the condition and I are in public, I would pretend that we are not related’ (*α*_White_ = .63, *α*_Asian_ = .77). The last item of the scale was removed from the composite score, it was coded in the opposite direction compared with all other items, this improved the Cronbach's alphas above .60. The mean of all items also yielded a reliable composite score for the full scale (*α*_White_ = .90, *α*_Asian_ = .95).

## RESULTS

Zero‐order correlations between all measures are reported in Table 1.^3^ In the White British sample, rating the person with depression as more dangerous of being violent was associated with lower closeness to the person with depression in the vignette, greater social distance towards people with depression in general, affective stigma by association as well as causal attributions to bad character, upbringing and God's will. Stigma by association was associated with lower closeness to the person with depression in the vignette, greater social distance towards people with depression in general and causal attributions to bad character, upbringing and God's will. In the South Asian sample, attributing the causes of depression to God's will was associated with lower closeness to the person with depression in the vignette. Affective, cognitive and behavioural stigma by association were also associated with greater social distance to the person with depression in the vignette.

**TABLE 1:** Zero‐order correlations between all measures, for White British (below the diagonal, *n* = 91) and for Asian British (above the diagonal, *n* = 46)

Measures	1	2	3	4	5	6	7	8	9	10	11	12
Causal attributions												
1. Character	–	.23	.39**	.37*	.00	−.08	.23	−.17	−.12	−.07	−.27	−.13
2. Brain	−.05	–	.26	−.13	.34*	−.13	.08	−.02	.05	.04	−.010	−.17
3. Upbringing	.40**	.05	–	.08	.23	.02	.22	−.01	.08	.10	.11	.09
4. Stress	.13	−.09	.31**	–	−.00	−.11	.02	.22	−.17	−.04	.09	−.02
5. Genetics	−.09	.41**	.23*	.01	–	.10	−.15	−.04	−.19	−.02	.02	−.09
6. God	.22*	.02	.09	−.01	.13	–	.10	−.46**	.05	−.07	.13	−.04
7. Dangerousness	.46**	−.01	.31**	.14	−.00	.22*	–	−.21	.02	−.08	−.15	.15
Discriminatory Potential												
8. Closeness	−.43**	.13	−.05	−.07	.21*	−.17	−.38**	–	−.22	−.07	−.11	−.25
9. Distance	.34**	−.173	.11	−.06	−.20	.17	.23*	−.27*	–	.76**	.61**	.67**
Stigma by Association												
10. Affective	.41**	−.04	.23*	.10	−.05	.23*	.23*	−.37**	.66**	–	.64**	.68**
11. Cognitive	.42**	.03	.30**	.20	.03	−.03	.10	−.14	.33**	.47**	–	.71**
12. Behavioural	.32**	−.02	.12	.02	−.13	−.01	.12	−.34**	.40**	.57**	.54**	–

*Note*: *p < .05, **p < .01 (two‐tailed).

### Preliminary analysis: Identification of depression

There were no significant differences for estimates of the likelihood with which the person in the vignette is experiencing a mental illness between White British (*M* = 4.16, *SD* = 0.93) and South Asians (*M* = 4.00, *SD* = 0.92), *t*(135) = 0.98, *p* = .328, Cohen's *d* = .18, and no significant differences for ratings of major depression between White British (*M* = 3.97, *SD* = 0.94) and South Asians (*M* = 4.02, *SD* = 0.75), *t*(135) = −0.35, *p* = .731, Cohen's *d* = −.06. In other words, both ethnic groups were able to correctly identify that the person in the vignette was described as having mental illness and major depression.

### Causal attributions of depression

To test for differences between White British and South Asians in their attributions of causes of depression, a multivariate analysis of variance (MANOVA) was carried out with the six causes as dependent measures. Cultural group was coded as White British = 0 and South Asian = 1. There was a significant multivariate effect (Pillai's trace *V* = .14, *F*[6, 130] = 3.40, *p* = .004, partial *η* ^2^ = .14). Univariate results revealed significant effects for the causes character, stress, God and brain. Results can be found in Table 2. South Asians reported greater attribution of the causes of the person's depression to the person's own bad character, to stressful circumstances in the person's life and to God's will. White British showed greater causal attributions to a chemical imbalance in the brain. There were no differences between White British and South Asians for the causes of genetics or the way the person was raised. Supporting H1, South Asians endorsed greater beliefs in spiritual as well as moral causes of depression, while White British endorsed greater beliefs in biological causes of depression. An independent *t*‐test revealed that South Asians also rated the person with depression in the vignette as higher in danger to be violent than White British, supporting H2a.

**TABLE 2:** Means (standard deviations) of all measures as a function of cultural group

Measures	White British (*n* = 91)	South Asian (*n* = 46)	*F*(1, 135)/*t*(135)	*p*	Partial *η* ^2^/Cohen's *d*
Causal Attributions					
Character	1.63 (0.75)	1.93 (0.77)	5.02	.027	.04
Brain	3.62 (0.88)	3.20 (1.07)	6.02	.015	.04
Upbringing	2.59 (0.92)	2.78 (1.05)	1.17	.281	.01
Stress	3.98 (0.83)	4.26 (0.68)	3.98	.048	.03
Genetics	2.90 (0.86)	2.67 (0.97)	1.97	.163	.01
God	1.29 (0.64)	1.70 (1.03)	8.22	.005	.06
Dangerousness	1.87 (0.75)	2.28 (0.86)	−2.91	.004	−.53
Discriminatory Potential					
Closeness	3.61 (0.95)	3.29 (0.70)	4.19	.043	.03
Distance	1.57 (0.97)	2.18 (1.40)	8.99	.003	.06
Stigma by Association					
Affective	1.29 (0.45)	1.80 (1.03)	15.79	<.001	.11
Cognitive	2.02 (1.39)	3.11 (2.11)	12.88	<.001	.09
Behavioural	1.75 (0.72)	2.17 (0.97)	8.19	.005	.06

*Note*: Causal attributions, dangerousness and willingness for closeness were measured on a 5‐point scale. Desire for social distance was measured on a 7‐point scale. Stigma by association was measured on a 9‐point scale.

### Discriminatory potential

To test for differences in willingness for closeness and desire for social distance to people with depression, a MANOVA was carried out with two dependent variables: closeness to the person with depression in the vignette and social distance towards people with depression in general (see Table 2). There was a significant multivariate effect (Pillai's trace *V* = .08, *F*[2, 134] = 5.40, *p* = .006, partial *η* ^2^ = .08). Univariate results revealed significant effects for both scales. South Asians reported a lower willingness for closeness to the person with depression in the vignette and a greater desire for social distance towards people with depression in general, than White British, supporting H2b.

### Stigma by association

To test for differences in affective, cognitive and behavioural stigma by association, a MANOVA was carried out (see Table 2). There was a significant multivariate effect (Pillai's trace *V* = .12, *F*[3, 133] = 6.06, *p* = < .001, partial *η* ^2^ = .12). Univariate results revealed significant effects for all three dimensions of stigma by association. South Asians reported greater negative affective, cognitive and behavioural stigma by the association of depression, than White British, supporting H2c.

### Mediation models

We computed two mediation analyses to assess whether perceptions of dangerousness and stigma by association mediated the relationship between cultural group and discriminatory potential (desire for social distance, willingness for closeness). Analyses were carried out using the PROCESS macro for SPSS (Hayes, 2022, Model 4). Cultural group was coded as White British = 0 and South Asian = 1. The significance of the mediation was tested using the bootstrapping procedure with 5000 resamples. For this analysis, the composite score for the full stigma by association scale was used (*M*_White_ = 1.84, *SD*_White_ = 0.66, *M*_Asian_ = 2.41, *SD*_Asian_ = 1.14). Belonging to the group of South Asians was significantly associated with greater perceptions of dangerousness (*B* = 0.41, *SE* = 0.14, *p* = .004) and stigma by association (*B* = 0.63, *SE* = 0.15, *p* < .001).

#### Closeness

**FIGURE 1:**
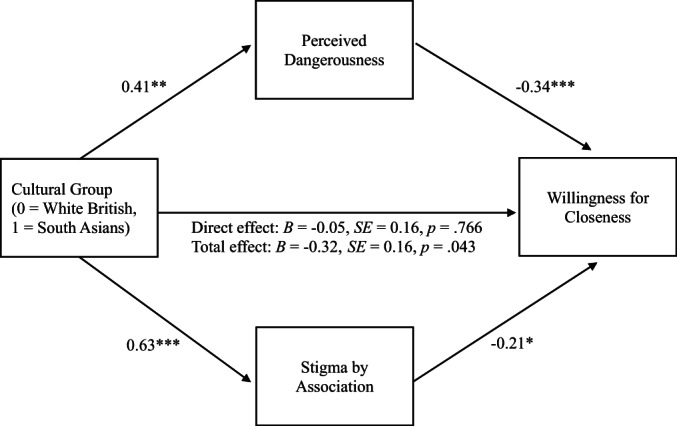
Perceptions of dangerousness and stigma by association as parallel mediators of the relationship between cultural group and willingness for closeness. Unstandardized coefficients are reported. Indirect effect perceived dangerousness: *B* = −0.14, *SE* = 0.06, 95% CI [−0.28, −0.03]. Indirect effect stigma by association: *B* = −0.13, *SE* = 0.06, 95% CI [−0.26, −0.03]. *p < .05, **p < .01, ***p < .001

Greater perceptions of dangerousness (*B* = −0.34, *SE* = 0.09, *p* = <.001) and greater stigma by association (*B* = −0.21, *SE* = 0.08, *p* = .011) were significantly associated with lower willingness for closeness to a person with depression. There were significant indirect effects through dangerousness (*B* = −0.14, *SE* = 0.06, 95% CI [−0.28, −0.03]) and stigma by association (*B* = −0.13, *SE* = 0.06, 95% CI [−0.26, −0.03]), and a significant total effect (*B* = −0.32, *SE* = 0.16, *p* = .043), but not direct effect (*B* = −0.05, *SE* = 0.16, *p* = .766) of cultural group on closeness through dangerousness and stigma by association. In sum, as hypothesized (H3), the relationship between cultural group and willingness for closeness was mediated by dangerousness and stigma by association (see Figure 1).

#### Social distance

**FIGURE 2:**
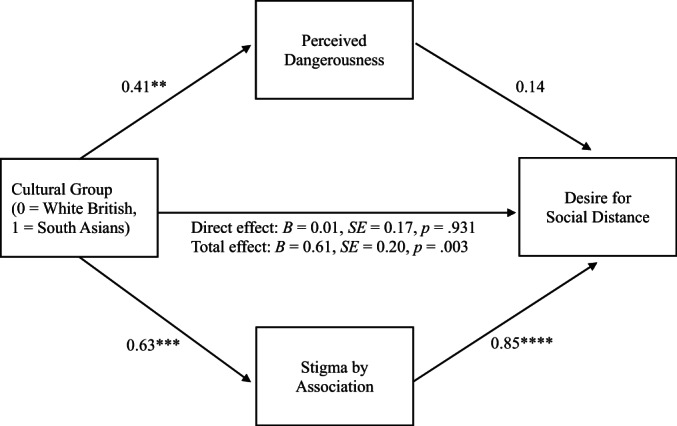
Stigma by association as a mediator of the relationship between cultural group and desire for social distance. Unstandardized coefficients are reported. Indirect effect perceived dangerousness: *B* = 0.06, *SE* = 0.05, 95% CI [−0.004, 0.17]. Indirect effect stigma by association: *B* = 0.54, *SE* = 0.18, 95% CI [0.22, 0.92]. **p < .01, ***p < .001, ****p < .0001

Greater stigma by association was significantly associated with greater desire for social distance towards people with depression (*B* = 0.85, *SE* = 0.09, *p* < .0001), the path from perceptions of dangerousness to social distance was not significant (*B* = 0.14, *SE* = 0.09, *p* = .118). There was a significant indirect effect through stigma by association (*B* = 0.54, *SE* = 0.18, 95% CI [0.22, 0.92]) but not through dangerousness (*B* = 0.06, *SE* = 0.05, 95% CI [−0.004, 0.17]), and a significant total effect (*B* = 0.61, *SE* = 0.20, *p* = .003), but not direct effect (*B* = 0.01, *SE* = 0.17, *p* = .931) of cultural group on social distance through dangerousness and stigma by association. In sum, the relationship between cultural group and desire for social distance was mediated by stigma by association (supporting H3) but not dangerousness (see Figure 2).

## DISCUSSION

Cultural differences in causal attributions and stigma towards mental illness is less well understood, particularly in regard to minority group members from Eastern cultures living in a dominant Western society. Our study expands the previous literature by comparing White British and South Asians living in the UK. Furthermore, while previous research that considered the role of culture has focused on public stigma towards people with depression, our study additionally considered the stigma of being associated with people with depression.

### Causal attributions of depression

Results revealed cultural differences in the beliefs about the aetiology of depression. South Asians attributed greater supernatural (God's will) and moral (bad character) causes to depression. White British endorsed greater biological beliefs (chemical imbalance in the brain) about the causes of depression. These findings support H1 and are in line with previous research suggesting greater supernatural attributions (Fogel & Ford, 2005; Lauber & Rössler, 2007; Mirza et al., 2019) and moral attributions (Krendl & Pescosolido, 2020; Raguram et al., 2004) in Eastern cultures and greater biological attributions in Western cultures (Dein & Bhui, 2013; Mirza et al., 2019). For example, Krendl and Pescosolido (2020) have demonstrated that individuals from Eastern cultures such as Bangladesh endorsed greater moral attributions than individuals from Western cultures such as the UK, in particular for depression. Mirza et al. (2019) have demonstrated that South Asians living in the UK endorsed greater supernatural beliefs about psychosis than White British. We are extending previous research by demonstrating that individuals from Eastern cultures living in a dominant society, that is South Asians living in the UK, endorse not only greater supernatural but also moral beliefs about the causes of depression than White British.

### Stigma towards depression

South Asians reported greater stigma towards people with depression than White British on several measures capturing public stigma and stigma by association. South Asians reported greater perceptions of dangerousness of people with depression (H2a) as well as a greater discriminatory potential in form of lower willingness for closeness and a greater desire for social distance towards people with depression (H2b) than White British. South Asians also indicated greater affective, cognitive and behavioural stigma by association (H2c). This is in line with previous research suggesting greater public stigma in Eastern cultures (Ahmed et al., 2020; Krendl & Pescosolido, 2020; Lauber & Rössler, 2007; Mirza et al., 2019) and expands these findings to stigma by association.

### Stigmatization processes

Our study also sheds light onto the stigma processes involved in the association between cultural group and stigma. Stigma by association mediated the relationship between cultural group and willingness for closeness and desire for social distance (H3). Perceived dangerousness was a mediator for willingness for closeness (H3). In other words, South Asians reported greater affective, cognitive and behavioural stigma by association, which in turn was associated with greater discriminatory potential and avoidance of people with depression. This is in line with previous findings in the Canadian context. Shamblaw et al. (2015) have shown that Canadian Asians reported greater social distance towards people with depression than Canadian Europeans. The belief that depression brings shame to the family, a form of stigma by association, mediated the relationship between cultural group and social distance.

A limitation of the current study is that we did not have more information about the participants from the South Asian community, such as whether they were born in the UK or elsewhere, or for how long they have been living in the UK. As acculturation processes and experiences such as discrimination may impact on their own mental health as well as perceptions of other people's mental health, an important avenue for future research would be to consider the background of South Asians in the UK in detail and how different backgrounds affect stigma.

### Implications

Our findings support previous arguments that stigma has greater social consequences in Eastern cultures, particularly for close relationships (Lauber & Rössler, 2007; Tabassum et al., 2000), and can be greater in cultural contexts characterized by interdependent self‐construals (He & Williams, 2021). Even though Eastern cultures may tend to be more delayed in seeking treatment, they likely could really benefit from it the most considering all the psychological burden they carry. Furthermore, causal explanations of depression and social support are important factors for help‐seeking, treatment and recovery from mental health and substance abuse disorders. For example, in individuals with substance abuse greater perceived social support is associated with greater self‐esteem, lower depression and anxiety, and higher quality of sleep. These effects were mediated by lower internalized stigma and shame (Birtel et al., 2017). Family members are a main source of social support for many individuals with mental illness for several reasons: Stigma is a major factor inhibiting professional help‐seeking due to fear of being stigmatized and excluded from society (Schomerus & Angermeyer, 2008; Schulze & Angermeyer, 2003; Wright et al., 2000), in particular South Asians underutilize mental health services (Fenton & Sadiq‐Sangster, 1996; Kapadia et al., 2018). Furthermore, access to mental health services is becoming increasingly difficult due to worldwide cuts (Keynejad et al., 2021). Additionally, in particular in Eastern cultures, the family plays a central role and stigma affects such close relationships to a greater extent than in Western cultures (He & Williams, 2021). This further underlines the importance of working towards reducing stigma and providing quality psychoeducation to family members. Therefore, interventions not only need to target mental illness stigma but also stigma by association with support family members. To test such interventions, validated measures that apply to Eastern cultures need to be developed (Abdullah & Brown, 2011). Future research should consider examining cultural differences between Eastern and Western cultures in a longitudinal study, to determine whether being associated with someone with depression causes greater social withdrawal in Eastern cultures than Western cultures.

## CONCLUSION

This study contributes to our understanding of the processes involved in the stigmatization of individuals with depression and individuals related to someone with depression, by considering the socio‐cultural context. Examining cultural differences in the perception of depression as well as stigmatization processes, and considering minority groups from Eastern cultures living in a dominant Western society, may inform culture‐sensitive interventions to support South Asians affected by depression as well as their family members and communities.

## AUTHOR CONTRIBUTIONS

**Michèle D. Birtel:** Conceptualization; data curation; formal analysis; methodology; supervision; writing – original draft; writing – review and editing. **Briana L. Mitchell:** Conceptualization; investigation; methodology; project administration; resources; writing – review and editing.

## CONFLICT OF INTEREST

All authors declare no conflict of interest.

## Data Availability

The data are available on request from the corresponding author.

## Footnotes

^1^
The symptoms were: depressed mood, feelings of worthlessness, loss of interest and pleasure, inability to concentrate, fatigue, insomnia, social withdrawal. Text of the vignette: *For the past two weeks Dev has been feeling really down. Dev wakes up in the morning with a flat heavy feeling that lasts throughout the whole day. Dev isn't enjoying things that normally seem fun or entertaining. In fact, nothing Dev seems to do seems pleasurable anymore. Even when good things happen, they don't seem to make Dev happy. It can be really hard, but Dev manages to push through each day. The smallest tasks are difficult to accomplish and it's hard to concentrate on anything. Dev feels out of energy and out of steam. However, no matter how tired Dev feels, it's always a challenge to fall asleep at night. Dev feels pretty worthless and very discouraged. Dev's family has noticed that these uncharacteristic changes have been going on for about the last month and that Dev has seemed to withdraw from them, as Dev just doesn't feel like talking*.

^2^
The data set was part of an MSc project that included other exploratory measures for the student.

^3^
Normality was tested for White British and South Asians separately. The data were generally normally distributed within the proposed range of −2 to +2 for skewness and − 7 to +7 for kurtosis (Byrne, 2010; Hair et al., 2010), apart from dangerousness for White British (skewness = 2.57), and affective and cognitive stigma by association for White British (skewness_affective_ = 2.11, skewness_cognitive_ = 2.10). The *F*‐test is robust against moderate deviations from normality (Montgomery, 1991) or even under non‐normality (Blanca et al., 2017; Schmider et al., 2010) and bootstrapping, such as the indirect effect in the Hayes' (2022) PROCESS mediation analysis, does not require normally distributed data.
